# Analysis of mRNA and lncRNA Expression Profiles of Breast Muscle during Pigeon (*Columba livia*) Development

**DOI:** 10.3390/genes13122314

**Published:** 2022-12-08

**Authors:** Yi Luo, Silu Hu, Peiqi Yan, Jie Wu, Hongrui Guo, Ling Zhao, Qianzi Tang, Jideng Ma, Keren Long, Long Jin, Anan Jiang, Mingzhou Li, Xuewei Li, Xun Wang

**Affiliations:** 1 Livestock and Poultry Multi-Omics Key Laboratory of Ministry of Agriculture and Rural Affairs, College of Animal Science and Technology, Sichuan Agricultural University, Chengdu 611130, China; 2 Animal Breeding and Genetics Key Laboratory of Sichuan Province, Institute of Animal Genetics and Breeding, Sichuan Agricultural University, Chengdu 611130, China; 3 College of Veterinary Medicine, Sichuan Agricultural University, Chengdu 611130, China

**Keywords:** pigeon, breast muscle, mRNAs, lncRNAs, development

## Abstract

The breast muscle is essential for flight and determines the meat yield and quality of the meat type in pigeons. At present, studies about long non-coding RNA (lncRNA) expression profiles in skeletal muscles across the postnatal development of pigeons have not been reported. Here, we used transcriptome sequencing to examine the White-King pigeon breast muscle at four different ages (1 day, 14 days, 28 days, and 2 years old). We identified 12,918 mRNAs and 9158 lncRNAs (5492 known lncRNAs and 3666 novel lncRNAs) in the breast muscle, and 7352 mRNAs and 4494 lncRNAs were differentially expressed in the process of development. We found that highly expressed mRNAs were mainly related to cell-basic and muscle-specific functions. Differential expression and time-series analysis showed that differentially expressed genes were primarily associated with muscle development and functions, blood vessel development, cell cycle, and energy metabolism. To further predict the possible role of lncRNAs, we also conducted the WGCNA and *trans*/*cis* analyses. We found that differentially expressed lncRNAs such as *lncRNA-LOC102093252*, *lncRNA-G12653*, *lncRNA-LOC110357465*, *lncRNA-G14790*, and *lncRNA-LOC110360188* might respectively target *UBE2B*, *Pax7*, *AGTR2*, *HDAC1*, *Sox8* and participate in the development of the muscle. Our study provides a valuable resource for studying the lncRNAs and mRNAs of pigeon muscles and for improving the understanding of molecular mechanisms in muscle development.

## 1. Introduction

The pigeon (*Columba livia*) was initially domesticated during the Neolithic period in the Middle East and/or Mediterranean regions [1,2,3]. As one of the most widely distributed poultry species in the world, pigeons were bred mainly for the production of utility (meat-type), sports, and as experimental or ornamental birds [4,5,6,7]. However, unlike other poultry, pigeons are the typical altrices, and their squabs need to be nurtured by their parents [8,9]. As the largest and most important tissue in poultry, skeletal muscle plays an essential role in body movement, protection, and metabolic regulation [10,11]. At the same time, the breast muscle is the largest muscle for pigeons, is essential for flight, and determines meat yield and quality for meat-type pigeons [12]. Previous studies have reported that young pigeons grow rapidly after hatching, particularly in the breast muscle [13]. So far, understanding of the molecular mechanisms of breast muscle growth in pigeons remains limited.

It is a complicated process for muscle development, which is regulated by many related coding or non-coding RNAs, transcription factors, and signaling pathways [14]. Myogenic progenitor cells undergo proliferation and differentiation during muscle development and form fused myotubes and myofibers [15]. There are embryonic and postnatal periods in the development of skeletal muscles [16]. Throughout the embryonic stages, skeletal muscle development is influenced by myoblast proliferation and the number of myofibers [10,17,18]. The primary causes of postnatal skeletal muscle development are fiber hypertrophy and the formation of new myonuclei inside preexisting myofibers [10]. Myocyte enhancer factor 2 (*MEF2*) family members and muscle regulatory factors (*Myf5*, *Myf6*, *MyoD*, and *MyoG*) are the major regulators of this complex process [19,20,21]. Additionally, many genes, such as *Pax3*, *Pax7*, *IGF-1*, and *PDK4*, participate in the regulation of skeletal muscle development [16,22].

At present, transcriptome sequencing (RNA-seq) has been utilized to study the genes and pathways during the development of muscles in main poultry, including chicken [23] and duck [10,24]. These studies mainly focused on investigating and analyzing the genes and pathways that influenced skeletal muscle growth and development [10,25,26] and played an important role in uncovering the potential mechanism of skeletal muscle development in poultry. Recently, Ding et al. first reported the mRNA expression of pigeon muscle during embryonic and postnatal early development [27]. However, studies regarding the mRNA and lncRNA expression profiles of muscle during the postnatal growth period of pigeons have not been reported.

In this study, the breast muscle transcriptomes of White-King pigeons over four stages (1 day, 14 days, 28 days, and 2 years old) were compared by RNA sequencing. We performed the differential expression, STEM, WGCNA, and trans/cis analyses to explore the function of mRNAs and lncRNAs in pigeon muscle development. Our studies will contribute to expanding the pigeon muscle lncRNA catalog and enhance understanding of the gene expression and regulation underlying the development in the skeletal muscle of pigeons.

## 2. Materials and Methods

### 2.1. Animals and Tissue Collection

The FengMao breeding farm (Mianyang, China) provided healthy White-King pigeons. Four developmental stages were assigned to the pigeons (each stage had 3 pigeon muscle samples): 1 day (1D), 14 days (14D), 28 days (28D), and 2 years old (2Y). Sodium pentobarbital infusion was used to euthanize the pigeons. The breast muscle was then gathered and swiftly frozen in liquid nitrogen. Finally, up to RNA extraction, all samples were kept in the ultra-freezer (Thermo Electron, Miami, FL, USA), at −80 °C.

### 2.2. Library Construction and Sequencing

Using Trizol reagent (Invitrogen, Carlsbad, CA, USA) and following the manufacturer’s instructions, the total RNA was isolated from frozen breast muscle samples. Agarose gel electrophoresis (1%) and an Agilent Bioanalyzer were used to evaluate the quality and quantity of the RNA (Agilent Technologies, Santa Clara, CA, USA). Poly-T oligo-attached magnetic beads (Invitrogen, Carlsbad, CA, USA) were used to purify RNA, and the RNA was then fragmented (approximately 300 bases) at 94 °C using a fragmentation buffer (Illumina, San Diego, CA, USA). Reverse transcription was performed using the resultant fragments as a template, and high-quality libraries were then sequenced (150 bp pair-end) using the Hiseq 4000 platform (Illumina, San Diego, CA, USA). To obtain high-quality data (clean data), the raw data were filtered by removing the reads containing the adapters, the reads containing more than 10% of the unknown nucleotides (N), and the reads containing more than 50% of low-quality bases (phred quality < 5).

### 2.3. Sequencing Analysis

STAR (v.2.6.0c) was used to align clean data to the pigeon genome (Cliv_1.0) [28], and the Cufflinks software was used to assemble the transcripts (v.2.1.1) [29]. For long non-coding RNA identification, firstly, transcripts that were clipped (first or last exons <15 bp), with short lengths (≤250 bp), or in a lowly abundance (FPKM < 0.1) were discarded as poorly assembled ones for each sample. Secondly, the transcripts were combined and compared to the reference genome using TACO software (v.1.0) [30], and those transcripts that were left unannotated by protein-coding genes (PCGs) were retained as putatively lncRNA transcripts. Thirdly, coding potential analyses were carried out using CPC2 (http://cpc2.cbi.pku.edu.cn/ accessed on 1 March 2021). Open reading frames were obtained using EMBOSS (version 6.5.7), and domain hits were verified using PfamScan (v.1.6) [31,32]. The domain hit of PCGs, and putative lncRNA transcripts were examined using Fisher’s exact test *p* > 0.05 or odds ratios < 10.0 were regarded as likely artifacts. The remaining transcripts were classified as long non-coding RNAs (CPC score > 0) based on the findings of coding potential analysis after deleting all the plausible domains.

### 2.4. Analysis of Differentially Expressed Genes

The expression level of mRNA or lncRNA was calculated by the transformed transcripts per kilobase million (TPM) using Kallisto (version 0.43.0) [33]. mRNAs with TPM > 0.5 and lncRNAs with TPM > 0.1 in at least two samples were considered to be expressed. Then, edgeR was used to detect differentially expressed (DE) mRNAs and lncRNAs [34], and the significant DE-mRNAs and DE-lncRNAs were screened with a cut-off criterion for FDR < 0.05 and |log2(FC)| > 1.

### 2.5. Time-Series Analysis

STEM software was used to analyze the enriched model profiles of DE-mRNAs and DE-lncRNAs [35]. The enriched model profiles with *p*-value < 0.05 were considered to be significantly enriched.

### 2.6. Functional Enrichment Analysis of mRNAs and lncRNAs

The Metascape website (http://metascape.org/ accessed on 28 April 2021) was used to conduct the Reactome Gene Sets, Gene Ontology Biological Processes (GO BP), and the KEGG pathway enrichment test. The WGCNA program created a weighted gene co-expression network to estimate the roles of DE-lncRNAs according to the expression levels of DE-mRNAs and DE-lncRNAs [36]. Metascape carried out functional analyses on the mRNAs from the top two co-expression modules in turn. In addition, we also performed the *cis* and *trans* analysis to gather the mRNAs within 100 kb and with strong correlations (|*r*| > 0.90 and *p* < 0.05) to DE-lncRNAs. Overlapping *trans* and *cis* results were also functionally analyzed.

## 3. Results

### 3.1. Summary of Sequencing Data

We constructed 12 RNA libraries from our pigeon samples and produced 277.28 million (23.11 million per sample on average) raw reads using 150-bp paired-end sequencing to study the transcriptome in breast muscles during pigeon development (Table 1). After the adaptors, unknown nucleotides, and low-quality reads were removed, 269.01 million (an average of 22.42 million per sample) clean reads remained for further analysis. The Q30 percentages ranged from 82.84% to 95.10%. Clean reads were mapped to the pigeon genome (Cliv_1.0), with a mapping rate above 90% for each sample.

### 3.2. Expression Profiles of mRNAs and lncRNAs in Breast Muscle

**Figure 1:**
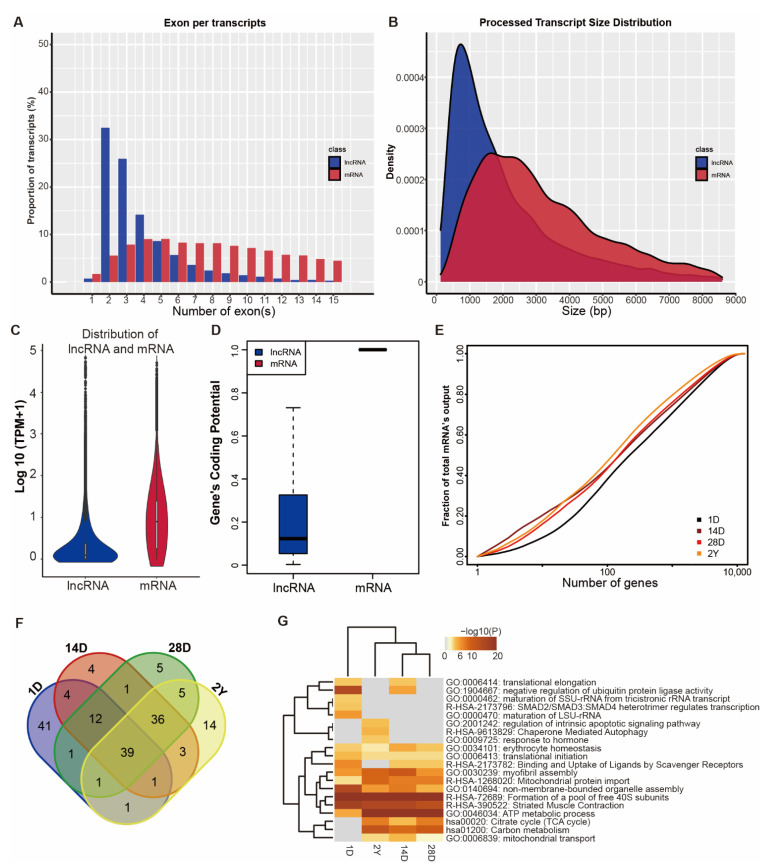
Genomic and transcriptional characterization of lncRNAs and mRNAs. (**A**,**B**) Exon number (**A**) and transcript size distribution (**B**) of lncRNAs and mRNAs. (**C**) The expression level and (**D**) coding potential of lncRNAs and mRNAs. (**E**) Cumulative distribution and (**F**) Venn diagrams of the top 100 highly expressed mRNAs in four developmental phases. (**G**) Function enrichment of the top 100 highly expressed mRNAs across four developmental stages. The stages of 1 day, 14 days, 28 days, and 2 years are represented by 1D, 14D, 28D, and 2Y.

By performing the transcriptome analysis, we identified a total of 12,918 unique mRNAs (Table S1, TPM > 0.5) and 9158 unique lncRNA (5492 known lncRNAs, 3666 novel lncRNAs, Table S2, TPM > 0.1), which were expressed in at least two samples. As shown in Figure 1A–D, we found that lncRNAs contained fewer exon numbers, shorter transcript lengths, lower expression levels, and lower coding potential (Figure 1A–D). Transcriptional complexity was seen to decrease slightly during the development stage, with the fraction of the top 100 highly expressed mRNAs ranging from 38% to 47%. (Figure 1E). Functional enrichment showed that highly expressed mRNAs (top 100) in each stage were enriched in muscle-specific and cellular functions-related terms (Figure 1F,G), such as myofibril assembly (GO:0030239), striated muscle contraction (R-HSA-390522), ATP metabolic process (GO:0046034), and mitochondrial protein import (R-HSA-1268020).

**Figure 2:**
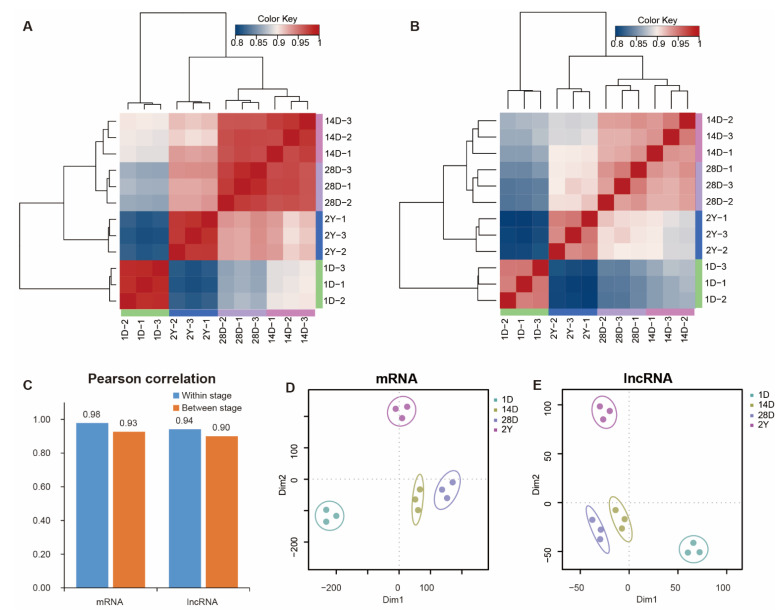
The Pearson’s correlation matrices and t-SNE analysis. (**A**,**B**) The mRNA (**A**) and lncRNA (**B**) correlation matrices for all samples, and the top and left panels show the sample trees based on the Pearson’s correlation. (**C**) The average correlation coefficients of mRNAs and lncRNAs between stages and within stages. (**D**,**E**) A two-way t-SNE plot of mRNAs (**D**) and lncRNAs (**E**) is shown based on expression profiles.

We used hierarchical clustering and Pearson’s correlation to analyze the expression of mRNAs and lncRNAs in each sample (Figure 2A,B and Figure S1). We found that mRNA and lncRNA expression data in pigeon breast muscles were mainly grouped into four clusters according to the developmental stages. At the same time, the 14D and 28D stages were clustered together and then clustered with the 2Y stage, while the major branch of the cluster was presented in 1D and other stages. Additionally, we also noted that mRNAs between the adjacent two stages had greater Pearson’s correlation coefficients than lncRNAs (Figure 2C). Similarly, the result of the t-SNE analysis indicated that the mRNA and lncRNA expression profiles of 12 samples appeared in a stage-specific manner (Figure 2D,E).

### 3.3. mRNA and lncRNA Differential Expression Analysis

**Figure 3:**
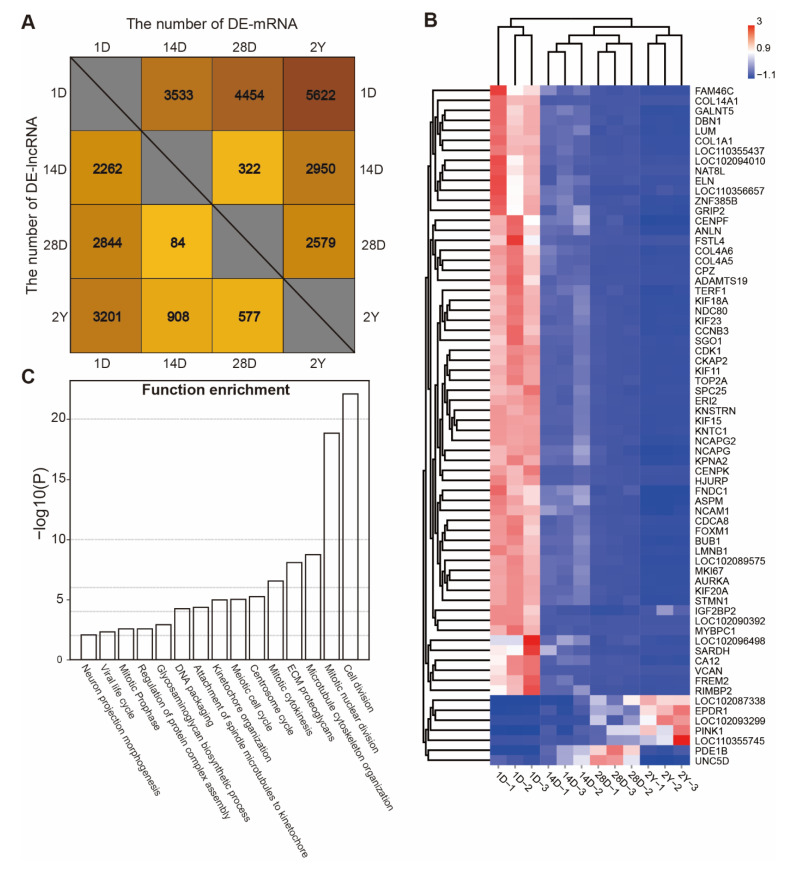
The identification and functional analysis of differently expressed genes. (**A**) The number of DE-mRNAs and DE-lncRNAs between stages (1D versus 14D, 1D versus 28D, 1D versus 2Y, 14D versus 28D, 14D versus 2Y, and 28D versus 2Y). (**B**) The expression heatmap of common differentially expressed mRNAs and lncRNAs. (**C**) The functional enrichment analysis was performed on the down-regulated expression of common DE-mRNAs.

By using |log2(fold change)| > 1 and the false discovery rate (FDR) < 0.05 as the cut-off criteria for pairwise comparisons, we were able to identify the DE-mRNAs and DE- lncRNAs at different stages. In total, we identified 7352 unique mRNAs and 4494 unique lncRNAs that were substantially and differently expressed during the pigeon’s development. These numbers represent 56.91% and 49.07% of the total number of mRNAs and lncRNAs we discovered, respectively (Tables S3 and S4). As shown in Figure 3A, the greatest number of DE-mRNAs and DE-lncRNA were 5622 and 3201 between the 1D and 2Y groups, while the minimum number of DE-mRNAs and DE-lncRNA were 322 and 84 between the 14D and 28D groups.

We also discovered that two DE-lncRNAs and 66 DE-mRNAs were differentially expressed in all the pigeon breast muscle development stages (Figure S2). As shown in Figure 3B, most of the common DE-mRNAs (61 of 66 mRNAs) had similar expression patterns. These genes were decreased and mainly related to the cell cycle and cell division (Figure 3C). The expression of EPDR1, PINK1, LOC102087338, LOC110355745, and LOC102093299 were gradually increased during development, while PDE1B and UNC5D were highly expressed in the 28D stage, indicating that these genes are related to the development of the muscle.

### 3.4. Analysis of DE-mRNA Expression Patterns and Functional Enrichment

**Figure 4:**
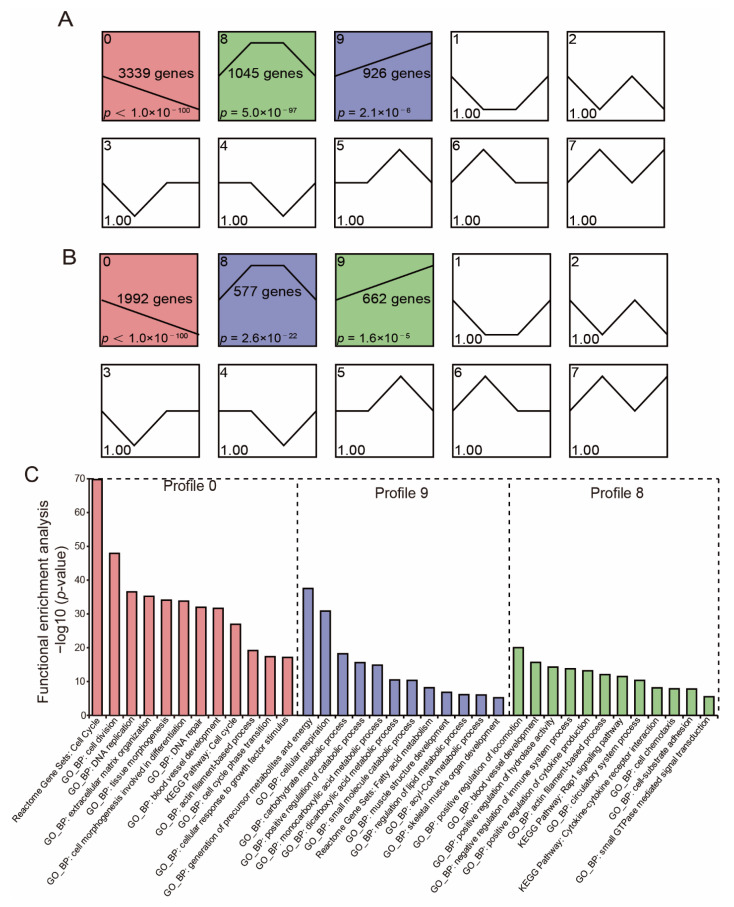
Time-course gene expression and functional enrichment analysis. (**A**,**B**) Time—series modules of DE-mRNAs (**A**) and DE-lncRNAs (**B**) are shown. The significantly enriched profiles are red, purple, and green colored (*p* < 0.05). The numbers in the upper-left corner of each pane denote the profile number; the numbers in the middle of each pane denote the number of genes in each profile; the numbers in the lower-left corner of each pane denotes the *p*-value of each profile. (**C**) Functional enrichment analysis of DE-mRNAs corresponding to significant profiles.

We used STEM to conduct a time-series analysis to examine the DE-mRNA and DE-lncRNA expression patterns in the breast muscles during pigeon development. As a result, 3231 lncRNAs and 5310 mRNAs were divided into three substantially enriched profiles, respectively (Figure 4A,B). Subsequently, we used the mRNAs in three profiles to perform the functional enrichment analysis. The genes in profile 9 were primarily enriched in the energy metabolic process (carbohydrate metabolic process, monocarboxylic acid metabolic process, positive regulation of catabolic process, regulation of lipid metabolic process, acyl-CoA metabolic process, fatty acid metabolism) and muscle development (muscle structure development, skeletal muscle organ development); the genes in profile 0 were primarily enriched in the cell cycle, cell division, DNA replication, tissue morphogenesis, and cellular response to the growth factor stimulus; the genes in profile 8 were primarily enriched in an actin filament-based process, blood vessel development, small GTPase mediated signal transduction, cell-substrate adhesion, and the positive regulation of cytokine production (Figure 4C).

### 3.5. The Functional Enrichment Analysis of DE-lncRNAs

**Figure 5:**
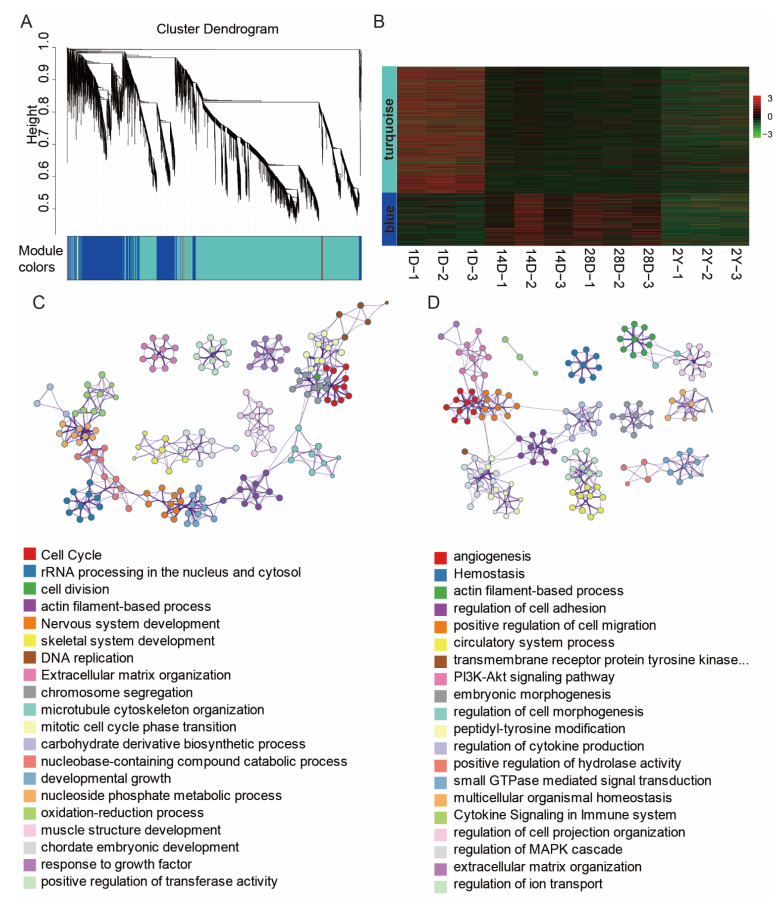
DE-lncRNA and DE-mRNA co-expression networks. (**A**) The co-expression network was constructed using the WGCNA method. The cluster dendrogram was produced using average linkage hierarchical clustering and is shown in the top panel. The bottom panel uses five colors to display the co-expression modules of lncRNAs and mRNAs. (**B**) Heat map showing the gene expression of the largest two co-expression network modules (blue and turquoise modules). (**C**,**D**) Functional categories of the mRNAs corresponding to turquoise and blue modules of lncRNAs.

It is still difficult to predict the function of lncRNAs because of their unique properties and the dearth of annotation resources. Here, we investigated the functional relationships between mRNAs and lncRNAs using co-expression analysis, which has been used to link lncRNAs to functionally annotated mRNAs. We constructed the co-expression network based on a non-redundant list of DE-mRNAs and DE-lncRNAs between neighboring stages (1D versus 14D, 14D versus 28D, and 28D versus 2Y) by WGCNA. In the end, five lncRNA and mRNA co-expression modules were discovered (Figure 5A). The lncRNAs and mRNAs in the top two modules accounted for 98.14% of the total number of genes in the five modules (Figure 5B). As shown in Figure 5C,D, the co-expressed genes in the turquoise module were primarily enriched in terms related to the cell cycle, cell division, developmental growth, and muscle structure development (Figure 5C); the co-expressed genes in the blue module were primarily enriched in terms related to angiogenesis, actin filament-based process, and the regulation of cell adhesion (Figure 5D).

**Figure 6:**
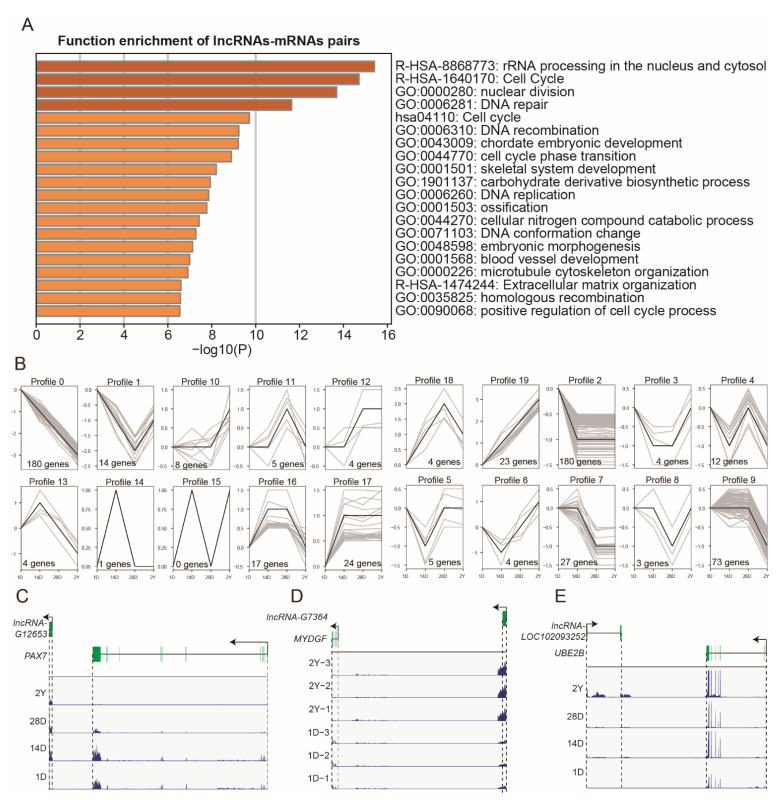
The *trans*/*cis* analysis of DE-lncRNAs and DE-mRNAs. (**A**) Functional enrichment analysis of DE-mRNAs that were significantly correlated and adjacent to the DE-lncRNAs. (**B**) The expression trends of DE-lncRNAs that were significantly correlated and adjacent to DE-mRNAs. (**C**–**E**) The loci and expression levels of the representative lncRNA-mRNA pairs: *G12653* and *PAX7* (1D, 14D, 28D, and 2Y), *G7364* and *MYDGF* (1D and 2Y), and *LOC102093252* and *UBE2B* (1D, 14D, 28D, and 2Y).

Additionally, in order to estimate the biological function of DE-lncRNAs, we carried out the trans/cis analysis to find the DE-mRNAs that were significantly correlated (|r| > 0.9, *p* < 0.05) and adjacent (within 100 kb) to the DE-lncRNAs. In total, 652 DE-mRNAs were highly correlated and concurrently located near 598 DE-lncRNAs (766 pairs, Table S5). These mRNAs were also associated with the cell cycle, skeletal system development, and blood vessel development (Figure 6A). Interestingly, we observed that the expression of these lncRNAs mostly showed a down-regulated trend in the process of breast muscle development. In contrast, 23 lncRNAs showed a gradually upregulated tendency, and some of their target mRNAs played an important role in muscle development [37,38,39] (Figure 6B). For example, the lncRNA-LOC102093252 targets UBE2B, lncRNA-G7364 targets MYDGF, lncRNA-G14041 targets PNPLA2, and lncRNA-G12653 targets PAX7 (Figure 6C–E). In addition to the above 23 lncRNAs, we also found that lncRNA-LOC110357465 targets AGTR2, lncRNA-G14790 targets HDAC1, and lncRNA-LOC110360188 targets SOX8 (Table S5 and Figure S3) with these genes were involved in muscle fiber composition and myogenesis [40,41,42].

## 4. Discussion

The muscle is one of the largest and most important tissues, which is crucial for body movement, protection, and metabolic regulation [10,11]. In livestock and poultry production, the mass of skeletal muscle directly correlates with meat production [43]. Skeletal muscle formation and growth are intricate processes, and gene expression is regulated by various regulatory molecules, such as lncRNAs and microRNAs (miRNAs) [14]. At present, numerous studies have partially explored the gene expression profiles of skeletal muscles at different growth stages in chickens [23], ducks [10,24], pigs [44], and cattle [45]. In addition, Ding et al. first reported the mRNA expression of pigeon muscles during embryonic and postnatal early development and discovered that the growth and development of pigeon skeletal muscles included several pathways such as PI3K/AKT/mTOR, AMPK, FAK, and thyroid hormone pathways [27]. However, studies regarding the mRNA and lncRNA expression profiles of muscles during the postnatal growth period in pigeons have not been reported. In this work, RNA-seq was used to identify the expression profiles of lncRNAs and mRNAs in the breast muscle of pigeons at four stages (1D, 14D, 28D, and 2Y). A total of 269.01 million clean reads were collected from 12 libraries, and we identified 12,918 mRNAs and 9158 lncRNAs (5492 known and 3666 novel lncRNAs). We discovered that lncRNAs had some different traits from mRNAs (fewer exon numbers, shorter transcript lengths, lower expression levels, and lower coding potential), which were consistent with previous studies in other species [46].

We observed that highly expressed mRNAs (top 100) were mainly related to essential cellular and muscle-specific functions. For example, *ACTN2* encodes sarcomeric α-actinin-2 proteins and constitutes the Z-line in skeletal muscle fibers [47]; *ACTA1* encodes skeletal muscle α-actin, which is an essential part of the contractile apparatus in the muscle [48]; the myosin light polypeptide 1 (*MYL1*) plays an essential role in the development of the skeletal muscle in embryonic, fetal and adult phases [49]. They are all highly expressed in the stages of muscle development and play an essential role in maintaining muscle-specific functions. According to our results, the expression profiles of lncRNAs and mRNAs were shown in a stage-dependent manner. The Pearson’s correlation and *t-SNE* assay indicated that lncRNAs and mRNAs have similar expression characteristics, and the major branch of the cluster was presented in 1D and other stages. This result might be related to the rapid breast muscle growth in the post-hatching of pigeons [13]. Moreover, Pearson’s correlation of lncRNAs between the adjacent stages was weaker than that for the mRNAs. This result was consistent with a previous study in the chicken liver [46], suggesting that lncRNAs might have a stronger temporal expression and show greater expression changes during the development process of pigeons.

Using a differential expression analysis, we discovered that most DE-lncRNAs and DE-mRNAs were downregulated during development. Only 66 DE-mRNAs and 2 DE lncRNAs were differentially expressed in all the pigeon breast muscle development stages. Interestingly, most of these common DE-mRNAs were also down-regulated during muscle development and were enriched in various cell division and cell cycle-related terms. This was consistent with the studies on skeletal muscle development: during embryonic stages, it relies on myoblast proliferation and the number of myofibers; during postnatal phases, it depends on fiber hypertrophy and the accumulation of new myonuclei inside preexisting myofibers [10,17,18]. Hence, cell division-related genes such as *BUB1* [50], *CDCA8* [51], *CDK1* [52], *CKAP2* [53], and *FOXM1* [54] could regulate cell proliferation and be downregulated during breast muscle development in postnatal phases. In addition, only three mRNAs and two lncRNAs gradually increased during development, indicating their important roles in muscle development. For example, *PINK1* is a mitochondrial kinase that participates in mitochondrial quality control and promotes cell survival [55]. D’Amora et al. reported that *PINK1* was highly expressed in skeletal muscles [56]. Likewise, Park et al. also reported that the *PINK1* mutants showed indirect flying muscles and dopaminergic neuronal degeneration along with locomotor abnormalities [57].

According to the results of expression patterns, we found that mRNAs and lncRNAs were dynamically expressed during the development of the breast muscle. Most DE-mRNAs and DE-lncRNAs have similar expression patterns, indicating that these genes may play similar roles in muscle development. The genes in profile 9 with an upregulated expression, such as *CAPN3*, *LDB3*, *TMOD4*, *LMOD3*, and *MYPN*, are all involved in muscle development and maturity [27,58,59,60,61,62]. *Myopalladin* (*MYPN*), a striated muscles–pecific gene, has been reported to have higher expression in postnatal than in the embryonic muscle of pigeons [27]. As a non-lysosomal cysteine protease, CAPN3 plays an important role in maintaining normal muscular function [62]. Kramerova et al. reported that the calcium release of myofibers in *CAPN3*-deficient muscles would be significantly reduced [62]. The protein of leiomodin-3 (LMOD3) is localized in sarcomere thin filaments, which is required for sarcomere integrity in myofibers, and *LMOD3*-deficient mice showed muscle atrophy and postnatal growth retardation [58]. In myofibers, carbohydrates and fat are the primary energy source for producing and sustaining muscle actions, while the basic energy pathways are stores of ATP and CP, anaerobic glycolysis, and oxidative phosphorylation [63]. Upregulated genes during development were mainly related to the carbohydrate metabolic process, positive regulation of the catabolic process, fatty acid metabolism, regulation of lipid metabolic process, and acyl-CoA biosynthetic process, which reflects the change in energy metabolism in the breast muscle. These results were in line with the physiologic role that the breast muscle plays during development.

In this study, we performed the WGCNA assay to predict the function of DE-lncRNAs. Similar DE-mRNA and DE-lncRNA expression patterns were grouped into a single module. Functional enrichment revealed that most of the co-expressed genes were involved in angiogenesis, the cell cycle, muscle structure development, and the actin filament-based process. This result is consistent with the *trans*/*cis* analysis, reflecting the critical role of lncRNAs in muscle development. As one of the E2 Ubiquitin-conjugating enzymes, *UBE2B* is essential for muscle protein homeostasis in catabolic conditions [38]; *Pax7* is crucial for controlling the expansion and differentiation of satellite cells during myogenesis, and its knockdown results in muscle atrophy and death [39]; the *AGTR2* gene is associated with the muscle fiber composition, athletic status and aerobic performance [40]; *HDAC1* regulates the transcriptional activity of *MyoD*, and influences the myogenic program [41]; *Sox8* negatively regulates the differentiation of skeletal muscles and inhibits myogenesis [42]. These genes were, respectively, targeted by *lncRNA-LOC102093252*, *lncRNA-G12653, lncRNA-LOC110357465*, *lncRNA-G14790*, and *lncRNA-LOC110360188*, suggesting that these lncRNAs may be crucial in controlling muscle development. Furthermore, earlier research revealed that the skeletal muscle was highly vascularized [64]. Skeletal muscle development and regeneration depend on the vascular system, which has a crucial role in physiological adaptation. In our results, we also found that angiogenesis-related DE-mRNAs were also targeted by particular DE-lncRNAs. These lncRNA-mRNA pairs reflect the important role of lncRNAs in skeletal muscle angiogenesis.

## 5. Conclusions

In summary, we investigated the mRNA and lncRNA expression profiles of pigeon breast muscles across four postnatal stages of pigeon development. In this study, we observed that skeletal muscle development is mainly associated with the cell cycle, energy metabolism, myogenesis, and angiogenesis pathways. Additionally, based on WGCNA and *trans*/*cis* analyses, our findings suggest that lncRNAs could be crucial for muscle growth. Our research enhanced our knowledge of the molecular processes involved in muscle development and offered an invaluable resource for studying lncRNA and mRNA in the pigeon muscle.

## Figures and Tables

**Table 1:** RNA-Seq data from breast muscle of White-King pigeon.

Sample	Raw Reads (M)	Clean Reads (M)	Raw Bases (Gb)	Clean Bases (Gb)	Mapped Ratio (%)	Q30 (%)
1D-1	23.31	22.77	6.99	6.83	92.36	92.64
1D-2	20.88	20.49	6.26	6.15	92.57	93.69
1D-3	23.18	22.70	6.95	6.81	91.91	92.41
14D-1	22.81	22.50	6.84	6.75	90.11	95.10
14D-2	23.84	23.25	7.15	6.97	91.24	91.98
14D-3	22.18	21.78	6.65	6.53	90.01	93.08
28D-1	23.09	22.53	6.93	6.76	90.88	91.42
28D-2	24.27	23.07	7.28	6.92	90.16	82.86
28D-3	24.07	23.46	7.22	7.04	91.26	92.94
2Y-1	23.89	22.79	7.17	6.84	90.36	82.86
2Y-2	24.78	23.69	7.43	7.11	90.42	82.86
2Y-3	21.01	20.01	6.30	6.00	90.65	82.84

## Data Availability

RNA-seq data are available via the Gene Expression Omnibus (GEO number GSE173122) of the NCBI (National Center for Biotechnology Information).

## Supplementary Materials

The following supporting information can be downloaded at: https://www.mdpi.com/article/10.3390/genes13122314/s1, Figure S1: The heat map based on the expression profiles of mRNAs (A) and lncRNAs (B); Figure S2: Venn diagrams of differentially expressed mRNAs and lncRNAs in four stages of development; Figure S3: The characteristics of gene expression for partial lncRNA-mRNA pairs; Table S1: mRNA expression data for pigeon breast muscle; Table S2: LncRNA expression data for pigeon breast muscle; Table S3: Differentially expressed mRNAs for pigeon breast muscle; Table S4: Differentially expressed lncRNAs for pigeon breast muscle; Table S5: LncRNA-mRNA pairs of DE-lncRNAs and DE-mRNAs.
