# Supplementary figures and images for: Analysis of mRNA and lncRNA Expression Profiles of Breast Muscle during Pigeon (Columba livia) Development
Source: Genes (Basel). 2022 Dec 8;13(12):2314. doi: 10.3390/genes13122314 (PMC9777807; doi:10.3390/genes13122314)

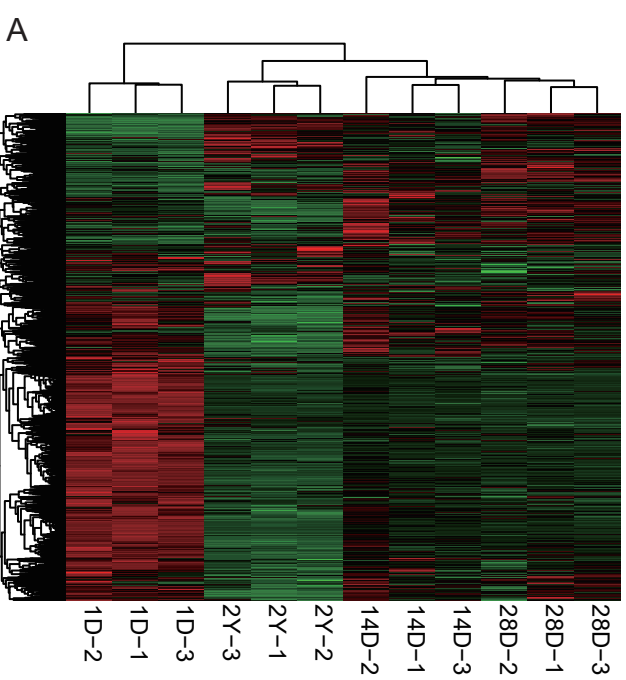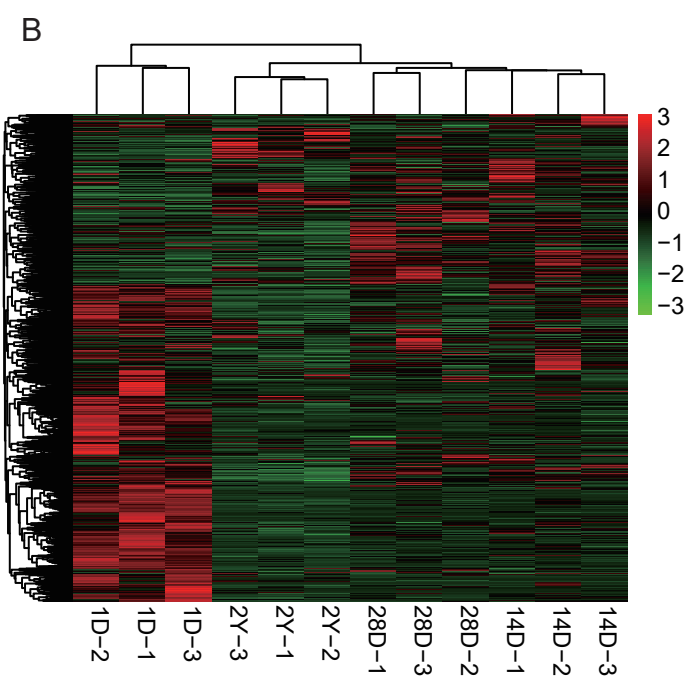

Supplement: Supplementary file 1 [file genes-13-02314-s001.zip › Figure S1.pdf]

A

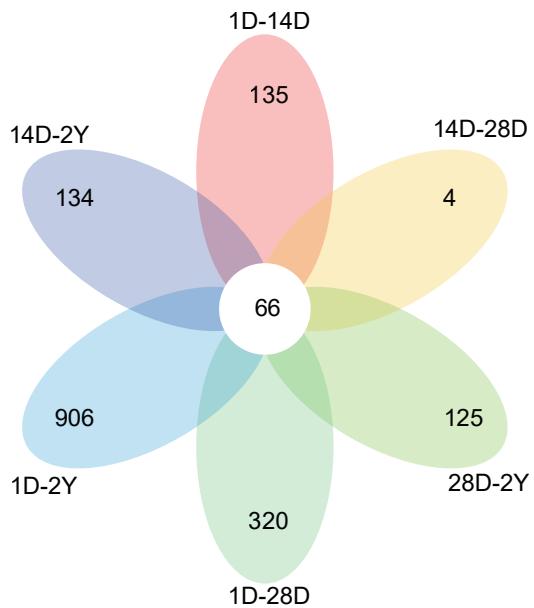

B

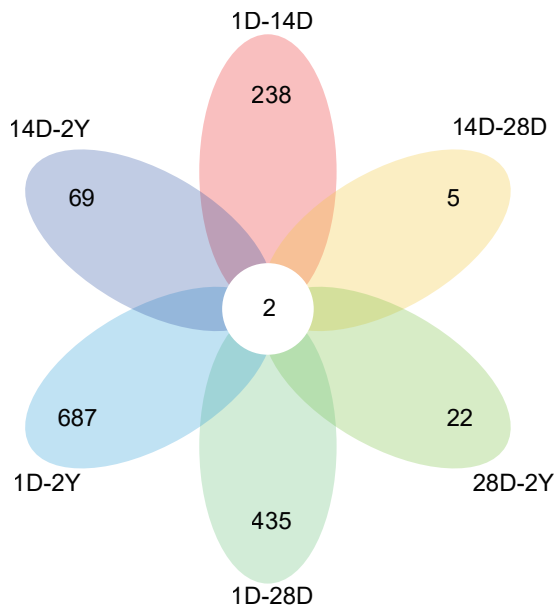

Supplement: Supplementary file 1 [file genes-13-02314-s001.zip › Figure S2.pdf]

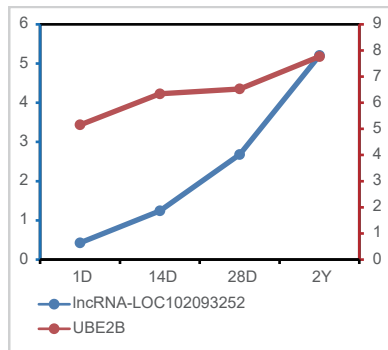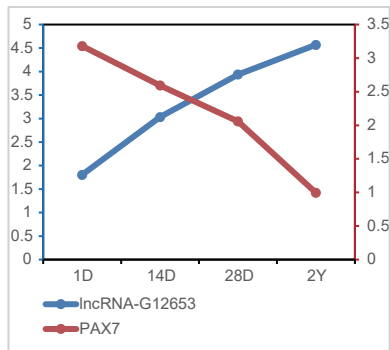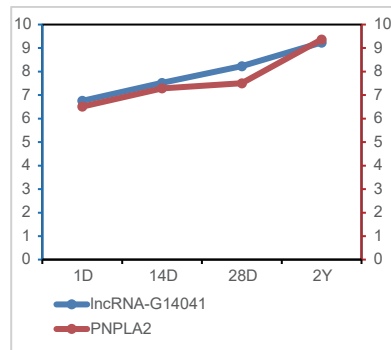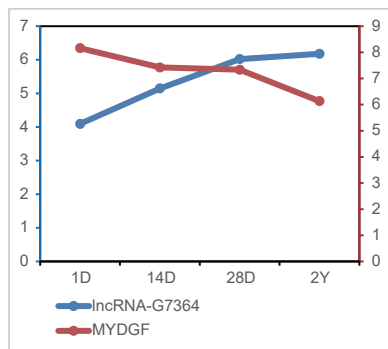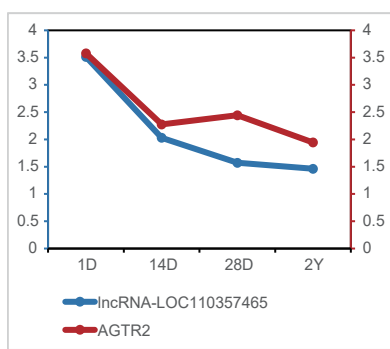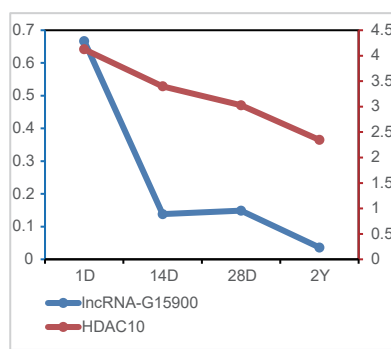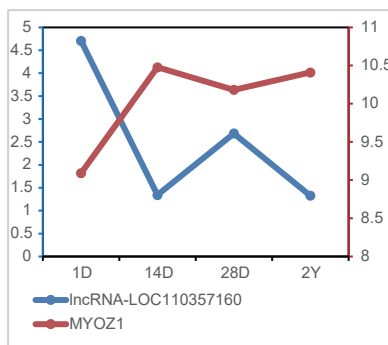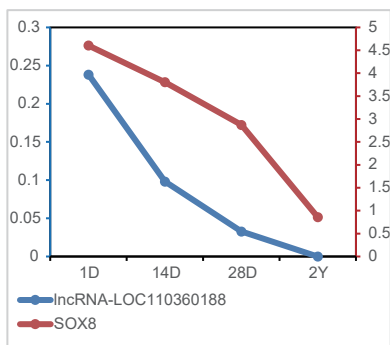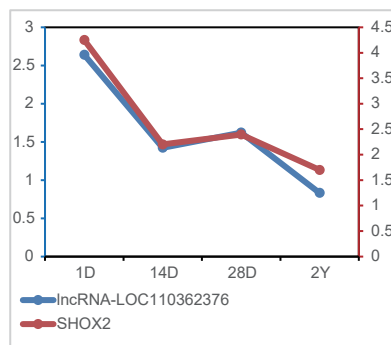

Supplement: Supplementary file 1 [file genes-13-02314-s001.zip › Figure S3.pdf]
